# Akt Signaling and Nitric Oxide Synthase as Possible Mediators of the Protective Effect of N-acetyl-L-cysteine in Prediabetes Induced by Sucrose

**DOI:** 10.3390/ijms25021215

**Published:** 2024-01-19

**Authors:** María Cecilia Castro, Hernán Gonzalo Villagarcía, Luciana Di Sarli Gutiérrez, Luisa González Arbeláez, Guillermo Schinella, María Laura Massa, Flavio Francini

**Affiliations:** 1 CENEXA—Centro de Endocrinología Experimental y Aplicada (UNLP—CONICET CCT La Plata, FCM, CEAS CICPBA), Calle 60 y 120, La Plata 1900, Argentina; mccastro@cenexa.org (M.C.C.); hvillagarcia@med.unlp.edu.ar (H.G.V.); ldisarli@med.unlp.edu.ar (L.D.S.G.); mlmassa@cenexa.org (M.L.M.); 2 CIC—Centro de Investigaciones Cardiovasculares (UNLP—CONICET CCT La Plata, FCM), Calle 60 y 120, La Plata 1900, Argentina; luisafarbelaez@med.unlp.edu.ar; 3 Facultad de Ciencias Médicas, UNLP, Calle 60 y 120, La Plata 1900, Argentina; schinell@med.unlp.edu.ar; 4 Instituto de Ciencias de la Salud, UNAJ-CICPBA (Av. Calchaquí 6200), Florencia Varela 1888, Argentina

**Keywords:** prediabetes, insulin resistance, unhealthy diet, IR-AKT-NOS pathway, N-acetyl-L-cysteine, glutathione reductase/oxidase

## Abstract

The aim of this work was to evaluate possible mechanisms involved in the protective effect of N-acetyl-L-cysteine (NAC) on hepatic endocrine-metabolic, oxidative stress, and inflammatory changes in prediabetic rats. For that, normal male Wistar rats (60 days old) were fed for 21 days with 10% sucrose in their drinking water and 5 days of NAC administration (50 mg/kg, i.p.) and thereafter, we determined: serum glucose, insulin, transaminases, uric acid, and triglyceride levels; hepatic fructokinase and glucokinase activities, glycogen content, lipogenic gene expression; enzymatic and non-enzymatic oxidative stress, insulin signaling pathway, and inflammatory markers. Results showed that alterations evinced in sucrose-fed rats (hypertriglyceridemia, hyperinsulinemia, and high liver fructokinase activity together with increased liver lipogenic gene expression and oxidative stress and inflammatory markers) were prevented by NAC administration. P-endothelial nitric oxide synthase (P-eNOS)/eNOS and pAKT/AKT ratios, decreased by sucrose ingestion, were restored after NAC treatment. In conclusion, the results suggest that NAC administration improves glucose homeostasis, oxidative stress, and inflammation in prediabetic rats probably mediated by modulation of the AKT/NOS pathway. Administration of NAC may be an effective complementary strategy to alleviate or prevent oxidative stress and inflammatory responses observed in type 2 diabetes at early stages of its development (prediabetes).

## 1. Introduction

Type 2 diabetes mellitus (T2D) is a prevalent disease increasing exponentially worldwide [1]. Its onset is preceded by a prediabetic state evinced by insulin resistance and impaired glucose tolerance. Its progression to T2D may be prevented or delayed by an intervening lifestyle or administrating specific drugs (metformin, sodium–glucose cotransporter 2 inhibitor, or glucagon-like peptide 1 receptor agonists) [2]. Modern societies are characterized by an enhanced consumption of calories and marked changes in the composition of meals (elevated ingestion of refined carbohydrates like sucrose or fructose) [3,4]. Several authors have suggested that this increase has paralleled the current epidemic of obesity and T2D [4,5]. Sucrose is a disaccharide of glucose and fructose. While a fructose-rich diet induces several features that are characteristic of metabolic syndrome, glucose does not. The differences between glucose and fructose effects are attributed to the distinct ways in which they are metabolized in the liver [6]. The liver is the main organ that metabolizes fructose and its hepatic metabolism accounts for at least half of the total and represents the focus of most concern. The gut, kidney, and muscles can also process fructose directly [7]. The fructose component of sugar sweeteners (nearly equivalent in sucrose and high fructose corn syrup) is considered a singularly harmful macronutrient [8].

Oxidative stress has been implicated as a major pathogenic cause in several illnesses, including metabolic diseases [9,10]. We have demonstrated that inflammation, insulin resistance, and oxidative stress, induced by a high fructose diet, constitute a pathological triad that could be effectively disrupted by mitigating endogenous oxidative stress [11]. N-acetyl-L-cysteine (NAC) has been employed in clinical practice for several decades. It has been used as a mucolytic agent and for the treatment of numerous disorders from paracetamol intoxication to chemotherapy-induced toxicity, HIV/AIDS, and psychiatric disorders [12].

NAC, a small molecule containing a thiol group with antioxidant capacity, is a well-known compound that, by acting through its reduced glutathione (GSH) enhancing effect [13], effectively counteracts, at least in part, the development of several oxidative stress-related dysfunctions, including peripheral IR in rodents [14,15,16] and humans [17,18], a dysfunctional liver [19,20,21], and obesity [22,23]. However, no studies have been focused on the application of NAC treatment to prevent the development of prediabetes, overall dysmetabolism, and liver malfunction induced by a sucrose administration and the possible signaling pathway involved. In consequence, this work is aimed at evaluating possible molecular mechanisms involved in the protective effect of NAC on hepatic endocrine-metabolic, oxidative stress, and inflammatory changes in prediabetic rats. In this case, we attempted to evaluate and report the involvement of insulin receptor-AKT-NOS pathway and glutathione reductase/oxidase expression in the protective effects of NAC on these alterations.

## 2. Results

### 2.1. Body Weight and Calories Intake

Calorie intake was similar in the three groups (control 21.5 ± 0.8; sucrose 23.6 ± 1.0; and sucrose/NAC group 23.7 ± 1.1 cal/100 g rat), showing a comparable bodyweight increase (control 101 ± 6.4, sucrose 106 ± 5.3; and sucrose/NAC 98 ± 6.5 Δg/rat).

### 2.2. Serum Measurements

Sucrose-treated rats present a significant increment in insulin levels and serum triglyceride concentration than control rats, with comparable glycemia in all groups (Table 1). Consequently, higher homeostasis model assessment of insulin resistance (HOMA-IR) and β-cell function by HOMA-β and lower liver insulin sensitivity indexes (LISI) values were recorded in sucrose rats, demonstrating that they portray lower insulin sensitivity both in peripheral tissues and in the liver (insulin resistance state) (Table 1). The administration of NAC for the last five days (Sucrose/NAC) drove the mentioned values to those recorded in control rats and prevented impairment of systemic and hepatic insulin sensitivity (Table 1). While aspartate aminotransferase (GOT) and alanine aminotransferase (GPT) values were no different in control and sucrose rats, GPT values were significantly lower in sucrose/NAC animals (Table 1). In a similar way, even when there were no differences in uric acid between control and sucrose rats, those animals treated with NAC showed significantly lower values of circulating uric acid (Table 1).

### 2.3. Oxidative Stress and Inflammation in the Liver

**Figure 1:**
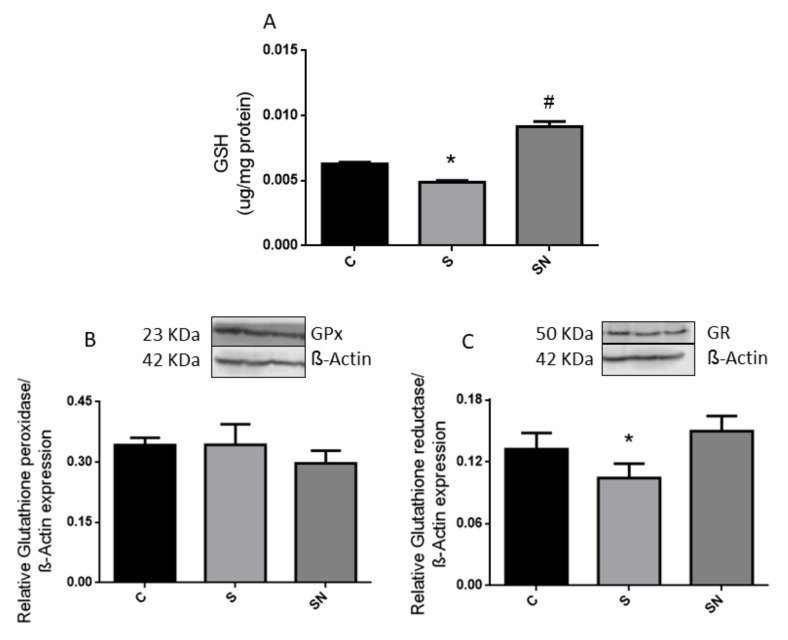
Liver reduced glutathione and hepatic protein content of glutathione peroxidase and glutathione reductase. Hepatic reduced glutathione (GSH) (**A**) and hepatic protein content of glutathione peroxidase (GPx) (**B**) and glutathione reductase (GR) (**C**). Black bars: control group (C rats), light grey bars: rats fed a sucrose-rich diet (S rats), grey bars: rats fed a sucrose-rich diet and N-acetyl cysteine (SN rats). Values are expressed as means ± SEM (n = 6 rats per group) * *p* < 0.05 compared to control group; # *p* < 0.05 compared to sucrose group. RE: relative expression.

The liver of sucrose-treated rats evinced a lower reduced glutathione (GSH) level compared to control rats (Figure 1A, *p* < 0.05). Accordingly, a significant reduction in glutathione reductase (GR) protein content was also recorded in sucrose animals (Figure 1C, *p* < 0.05). No differences were detected in the protein content of glutathione peroxidase (GPx) (Figure 1B).

**Figure 2:**
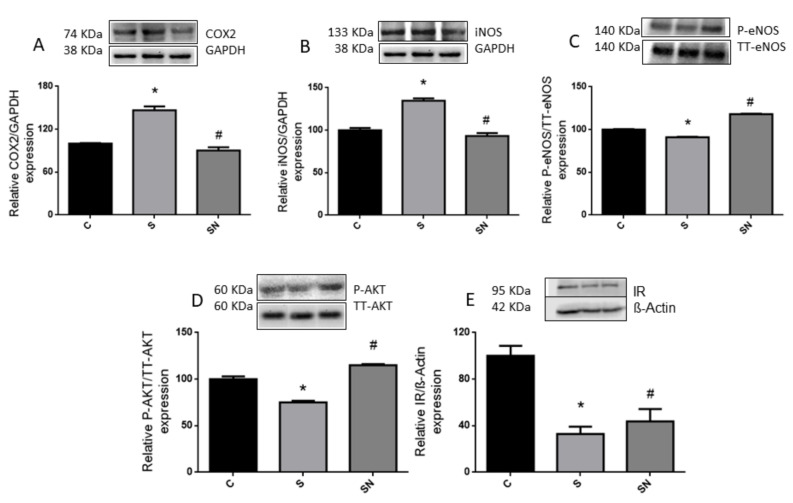
Liver protein content of COX2, iNOS, P-endothelial nitric oxide synthase (P-eNOS) P-AKT, insulin receptor (IR). Hepatic protein content of COX2 (**A**), iNOS (**B**), P-endothelial nitric oxide synthase (P-eNOS) (**C**), P-AKT (**D**), insulin receptor (IR) (**E**). Black bars: control group (C rats), light grey bars: rats fed a sucrose-rich diet (S rats), grey bars: rats fed a sucrose-rich diet and N-acetyl cysteine (SN rats). Values are expressed as means ± SEM (n = 6 rats per group) * *p* < 0.05 compared to control group; # *p* < 0.05 compared to sucrose group.

Sucrose treatment induced an increment in cyclooxygenase-2 (COX-2) protein content, an alteration prevented by NAC treatment (Figure 2A, *p* < 0.05). No changes were detected at mRNA levels between treatments. In parallel, sucrose treatment also induces a significant increment in inducible nitric oxide synthase (iNOS) protein content. NAC was able to restore these values to near the control ones (Figure 2B, *p* < 0.05).

### 2.4. Hepatic Metabolic Alterations

**Figure 3:**
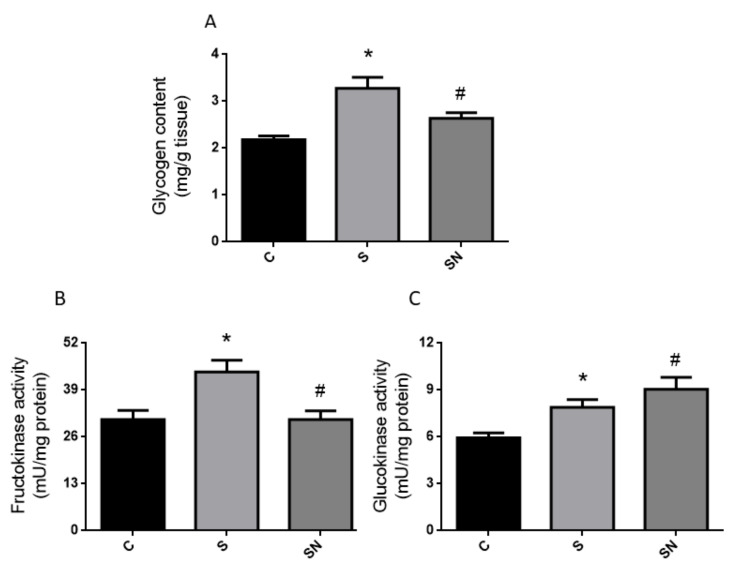
Hepatic glycogen content and fructokinase and glucokinase activities. Liver glycogen content (**A**), fructokinase activity (**B**), glucokinase activity (**C**). Black bars: control group (C rats), light grey bars: rats fed a sucrose-rich diet (S rats), grey bars: rats fed a sucrose-rich diet and N-acetyl cysteine (SN rats). Values are expressed as means ± SEM (n = 9 rats per group) * *p* < 0.05 compared to control group; # *p* < 0.05 compared to sucrose group.

**Figure 4:**
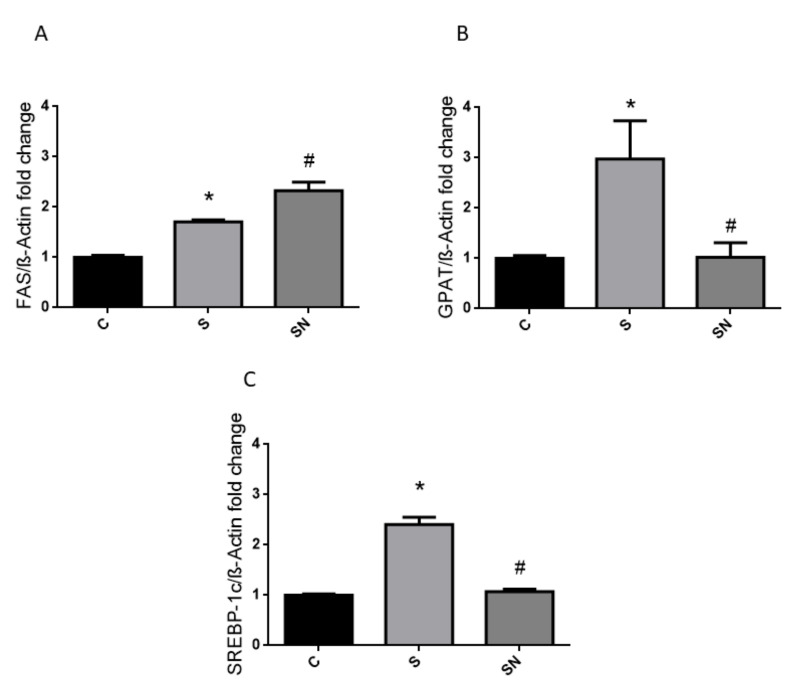
Liver gene expression for lipid metabolic pathways. mRNA levels of *fatty acid synthase (FAS)/ β-Actin* (**A**), mRNA levels of *GPAT/β-Actin* (**B**), mRNA levels of *SREBP-1c/β-Actin* (**C**). Black bars: control group (C rats), light grey bars: rats fed a sucrose-rich diet (S rats), grey bars: rats fed a sucrose-rich diet and N-acetyl cysteine (SN rats). Values are expressed as means ± SEM (n = 6 rats per group) * *p* < 0.05 compared to control group; # *p* < 0.05 compared to sucrose group. RE: relative units.

Regarding liver carbohydrate metabolism, the sucrose treatment induced higher glycogen content in the liver, which was reverted by the antioxidant (Figure 3A). On the other hand, fructokinase and glucokinase (hepatic glucose sensor) activities were significantly (Figure 3B,C, *p* < 0.05 vs. control) higher in sucrose-treated rats. The increase in fructokinase activity was fully prevented by NAC therapy. Finally, fatty acid synthase (*FAS*), sterol regulatory element-binding proein-1c (*SREBP-1c*), and glycerol-3-phosphate acyltransferase (*GPAT*) liver gene expression, increased in sucrose-fed rats, were normalized to the control values in rats treated with NAC for *SREBP1c* and *GPAT* expressions (Figure 4A–C).

### 2.5. Insulin Signaling Pathway

The results showed lower IR levels and P-eNOS:eNOS protein ratio, together with a reduction in P-AKT in sucrose-fed rats. Interestingly, NAC treatment was highly effective to significantly (*p* < 0.05) enhance both protein ratios (P-eNOS:eNOS and P-AKT:AKT), but was not able to improve the IR protein content (Figure 2C–E).

## 3. Discussion

It has been previously shown that normal rats fed refined carbohydrates for 21 days developed several generalized metabolic and endocrine disorders [24,25]. The underlying mechanisms responsible for such detrimental effects are related to a pathological triad: insulin resistance, oxidative stress, and inflammation [11]. Such alterations include significant changes in carbohydrate and lipid metabolism that would channel liver metabolites preferentially to energy storage rather than to mitochondrial oxidation [24,26,27].

Interestingly, taken together, the above-mentioned alterations resemble those described in human metabolic syndrome. Consequently, the sucrose-fed rats constitute a suitable model for studying pharmacological interventions at early stages of diabetes development (prediabetes).

In the current experiments, we demonstrated that induced insulin resistance evinced by a higher circulating insulin level, together with normoglycemia and in consequence creating higher HOMA-IR and lower LISI indexes, triggered by sucrose, are prevented by NAC administration. Sucrose-fed rats also evinced hypertriglyceridemia, enhanced liver lipogenic gene expression and carbohydrate dysmetabolism (increased glucokinase, fructokinase, and higher glycogen content), down regulation of the antioxidant defense system (decreased GSH levels together with reduced GR expression), and an inflammatory state (high COX-2 and iNOS protein content) and altered insulin pathway response. NAC treatment ameliorates insulin resistance and liver metabolic disturbances, oxidative stress, and inflammation. The results demonstrated that NAC effects could be mediated by enhancing GSH synthesis via GR and a parallel return to basal conditions in the P-AKT/AKT and consequently P-eNOS/eNOS ratios.

AKT-dependent phosphorylation at Ser is an important mechanism for enhancing nitric oxide synthesis by eNOS [28,29,30]. Therefore, it is interesting to speculate that the enhancement of the P-eNOS level shown by NAC-treated animals could be a positive feature related to a possible preventive effect on insulin-resistant states. In accordance with NAC improvement of insulin sensitivity, Michlin et al. [31] recently demonstrated that the maternal administration of NAC during pregnancy and lactation improves glucose tolerance of offspring at adulthood. Isolated islets of NAC-treated offspring (6 weeks old, before high fat diet feeding) had an increased efficacy of glucose-stimulated insulin secretion and a higher resistance to oxidative damage. Following high fat diet feeding, glucose tolerance and insulin sensitivity of NAC-treated offspring were improved.

In the current experiments, fructokinase activity, a marker of liver metabolism clearly displaced to lipogenesis, was enhanced. This enzyme has a key role in channeling carbons into lipogenic pathways, bypassing the regulatory steps of glycolysis. NAC treatment restored blank values, thereby suggesting a new site of action of this antioxidant. Interestingly, the activity of liver glucose sensor, glucokinase, increased in S rats, was not modified by NAC treatment, while protein returned to basal values, thus suggesting a different time course for protein and activity regulation. Finally, the liver glycogen deposit, clearly enhanced in sucrose-fed animals, was also restored to normal in NAC-administered rats, thereby suggesting a general improvement of carbohydrate metabolism.

Dyslipemia and fatty liver are also common components of metabolic syndrome; in fact, our prediabetic sucrose-fed rats showed high circulating triglyceride levels as well as enhanced expression of liver lipogenic genes (*SREBP-1c* and *GPAT*). These parameters were fully reverted by NAC administration. In a model of liver damage induced by long-term CCl4 administration, Otrubová et al. [32] demonstrated that NAC significantly decreased levels of cholesterol, bile acids, and bilirubin in plasma and triacylglycerols in liver, all of them elevated by impairment with CCl_4_. Taken together, these results suggest that NAC can also modulate lipid metabolism.

In a rat model, the administration of NAC has both gastro-protective and anti-inflammatory effects promoting anti-ulcerative action. In gastric tissues, NAC administration decreased the level of lipid peroxidation and activity of catalase, which were increased by induced ulcers. Moreover, NAC increased the GSH level and superoxide dismutase and glutathione S-transferase activities, which decreased in ulcerous stomach tissues [33]. Dludla et al. [34] wrote an exhaustive review supporting the ameliorative effects of NAC against metabolic dysregulations, chronic immune activation, and OS associated with obesity in vitro and in vivo models. In accordance with these results, NAC treatment restored the GSH content, with an increment in the GR protein levels, parameters which were altered by the treatment with sucrose. However, no changes were recorded regarding the GPx protein level.

It is known that the inhibition of PI3K/AKT/mTOR pathway may lead to autophagy or apoptosis. It has been also demonstrated that ROS accumulation leads to autophagy via the inhibition of AKT/mTOR phosphorylation; NAC markedly reversed drug-induced effects on the PI3K/AKT/mTOR cascade, as well as on the levels of autophagy-related proteins, probably due to NAC scavenger properties. This inhibition seems to be mediated by nuclear translocation of TFEB, as well as changes in key molecules of the AKT/mTOR signaling pathway and downstream autophagy [35]. Interestingly, using a murine model of prediabetes, Maiztegui et al. [36], demonstrated that fructose induced an increase in both VMP1 and LC3-II proteins, suggesting that autophagy is activated in injured pancreatic β-cells. Taken together, both results suggest that the redox state and oxidative stress triggered by unhealthy diets could be related to autophagy and that NAC modulation of oxidative stress could modify nuclear translocation of key factors. However, the hypothesis that this mechanism is also operative in the liver of sucrose-fed rats needs further research.

**Figure 5:**
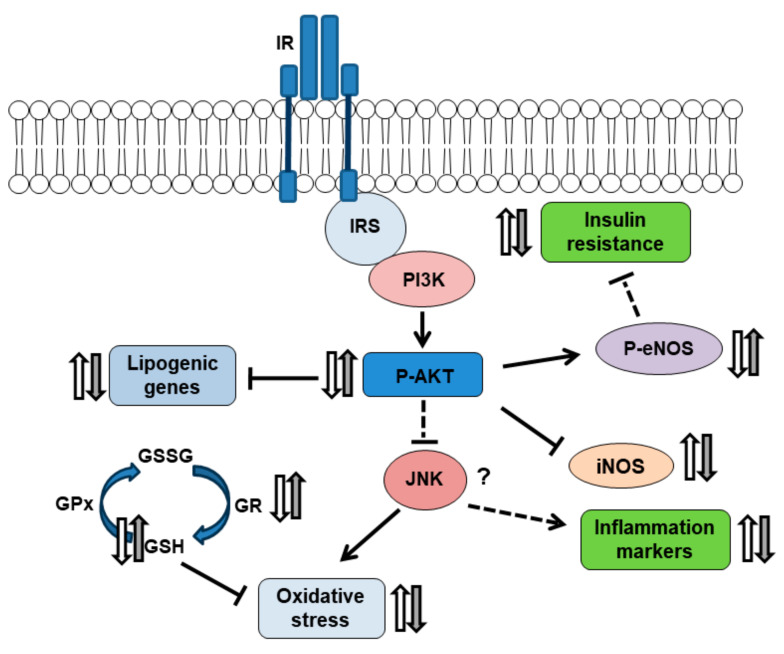
Schematic diagram representing the possible mechanism of action of NAC. Increased lipogenic gene expression, inflammatory markers, and oxidative stress measured in sucrose-fed animals (white arrows) were reversed by NAC administration (grey arrows), modulating Akt/eNOS and GPx/GR pathways. IR: insulin receptor; IRS: insulin receptor substrate; PI3K: phosphatidil-inositol-3-kinase; GSSG: oxidate glutathione; JNK: Jun kinase.

The sucrose-rich diet also triggered a hepatic inflammatory response evinced by enhanced expression of COX-2 and iNOS protein. NAC administration reduced inflammatory processes triggered by unhealthy diets. Consequently, dietary administration of NAC resulted in an effective strategy to alleviate or prevent oxidative stress and inflammatory responses observed in our prediabetic rats, probably mediated by modulation of the AKT/NOS pathway (Figure 5).

Although extrapolation from a murine surrogate model to clinical research must be justified by means of a variety of theoretical and experimental considerations, it is interesting to consider that these findings have a potential translational significance for a possible therapeutic tool to treat diabetes at an early stage of development (prediabetes) and merit further investigation to validate the results in human subjects.

## 4. Materials and Methods

### 4.1. Chemicals and Drugs

Chemicals and drugs of the purest available grade including NAC were provided by Sigma Chemical Co. (St. Louis, MO, USA). Primary antibodies anti-P-AKT (reacting with Ser473), anti-AKT, anti-Insulin-receptor (anti-IR), anti-fructokinase (anti-fructokinase), anti-glutathione-peroxidase (anti-GPx), and anti-glutathione reductase (anti-GR) were obtained from Santa Cruz Biotechnology, Inc. (Santa Cruz, CA, USA; catalog number 6040S, 9272,sc-711, sc-50029, sc-133160, and sc-133245, respectively). Anti-COX-2 from CAYMAN Laboratories (Ann Arbor, MI, USA catalog number 160106), anti-iNOS, and anti-eNOS were obtained from Sigma (St. Louis, MO, USA; catalog number N7782). Anti-P-eNOS (Ser 1177) was provided by Cell Signaling Laboratory (Danvers, MA, USA; catalog number N3893); anti-glucokinase antibody (sheep anti-GST-glucokinase fusion protein antibody) was kindly provided by Dr. Mark Magnusson (Vanderbilt University, TN, USA). This antibody, another from Santa Cruz Biotechnology Inc. (Santa Cruz, CA, USA; glucokinase-N-19: sc:1980), and anti-GAPDH from Millipore (Carlsbad, CA, USA; catalog number 92590) were also provided. Finally, a secondary antibody anti-rabbit IgG Peroxidase (developed in goats) was obtained from Sigma (St. Louis, MO, USA; catalog number A9169).

### 4.2. Animals

Normal male Wistar rats (150–180 g) were in metallic cages at 23 °C with a fixed 12 h light–dark cycle (06:00–18:00 h). Animals were divided into three experimental groups: standard commercial diet ad-libitum and tap water (control group), the same diet plus 10% sucrose in the water (Sucrose group), and the sucrose group injected with NAC (50 mg/kg, i.p.) (Sucrose/NAC) during the last five days of treatment (from day 16 to 21). The control and sucrose animals were injected with the same volume of saline buffer. Water intake was measured daily, and individual body weight as food intake were recorded weekly. This procedure was replicated three times (total, 12 animals per group). After twenty-one days of sucrose treatment, blood samples from 4 h fasted animals were drawn from the retroorbital plexus under light halothane anesthesia and collected into heparinized tubes to measure blood glucose, triglyceride, and immunoreactive insulin levels. Afterwards, the animals were sacrificed by decapitation and a portion of the median lobe of the liver was removed to perform all the assays. When the assays were not performed immediately, the lobe was quickly immersed in liquid nitrogen and thereafter stored in a deep freezer at −80 °C; all enzyme activities were measured within a week. Animal experiments and handling were performed according to the “Ethical principles and guidelines for experimental animals” (3rd Edition 2005) of the Swiss Academy of Medical Sciences [37]. All the protocols were approved by the Animal Welfare Committee (CICUAL) of the La Plata School of Medicine, UNLP (P05-04-2017).

### 4.3. Serum Measurements

Glucose was measured with the glucose-oxidase GOD-PAP method (Roche Diagnostics, Mannheim, Germany). Plasma levels of triglycerides, uric acid, and transaminases (aspartate aminotransferase-GOT and alanine aminotransferase-GPT) were assayed by commercial (enzymatic-colorimetric) kits (Wiener Lab., Rosario, Santa Fe, Argentina). Circulating immunoreactive insulin was determined by a previously described specific radioimmunoassay [38].

Glycemia and insulin values were used to estimate peripheral IR by homeostasis model assessment of insulin resistance (HOMA-IR) (insulin × glycemia/22.5) [39] and β-cell function by HOMA-β [(20 × insulin/glycemia) − 3.5]. Liver insulin sensitivity index (LISI) was calculated by the following formula: k/(fasting plasma insulin) × fasting glycemia, where k = 22.5 × 18 (insulin/glycemia) [40]. Insulin and glycemia were expressed as μUI/mL and mM, respectively.

### 4.4. Liver Reduced Glutathione (GSH)

Hepatic oxidative stress marker GSH was determined as described elsewhere [24] and measured spectrophotometrically at 414 nm. Results were expressed in μmol of GSH per g of tissue.

### 4.5. Total Liver RNA Isolation and mRNA Expression Levels by Real Time PCR (qPCR)

A 100 mg liver piece was used for total RNA isolation using TRIzol Reagent (Gibco-BRL, Rockville, MD, USA) as described in a previous report [41]. The integrity and quality of RNA isolated was checked by agarose-formaldehyde gel electrophoresis and by measuring the 260/280 nm absorbance ratio. DNA contamination was avoided by using DNase I digestion reagent (Gibco-BRL). cDNA was obtained by reverse transcription-PCR using Super Script III (Gibco-BRL) and total RNA (50 ng) as a template.

We utilized a Mini Opticon Real-Time PCR Detector Separate MJR (BioRad, Hercules, CA, USA) and SYBR Green I as fluorescent dye for qPCR reactions. For this purpose, 10 ng of cDNA was amplified using FastStart SYBR Green Master mix (Roche Diagnostics GmbH, Mannheim, Germany) with 40 cycles (denaturation at 95 °C for 30 s, annealing at 65 °C for 30 s, and extension at 72 °C for 45 s). Negative controls consisted of replacing samples with the same volume of water.

Specific oligonucleotide primers (obtained from Invitrogen) are shown in Table 2. Amplicons were designed in a size range of 90 to 250 bp, with β-actin used as housekeeping gene. Results are shown as relative to β-actin gene expression. Reaction specificity was checked by melting curve analysis. Data were calculated as relative gene expression after normalization to the β-actin housekeeping gene using Qgene96 4.4.0 and LineRegPCR (https://mybiosoftware.com/linregpcr-analysis-quantitative-pcr-data.html, accessed on 10 August 2023) software as described elsewhere [42,43,44]. Once obtained, these values were then expressed as the fold change compared to the marker of interest/β-actin gene expression on the control group (considered as 1).

### 4.6. Western Blot Analysis

Immunodetection of COX-2, GPx, GR, IR, iNOS, eNOS/P-eNOS, AKT/P-AKT, and GAPDH proteins was performed in liver homogenates from each experimental group. Protein concentration was quantified by Bio-Rad Protein Assay kit. Thereafter, dithiothreitol and bromophenol blue were added (final concentrations 100 mM and 0.1% *w*/*v*, respectively). Aliquots of 50–100 μg of whole protein were placed in reducing 10% (*w*/*v*) SDS-PAGE and electroblotted to polyvinylidene difluoride membranes. GAPDH density was used to normalize protein content. Non-specific binding sites of membranes were blocked by overnight incubation with non-fat dry milk at 4 °C. Protein identification and quantification were performed with specific primary antibodies against COX-2, GPx, GR, IR, iNOS, eNOS, P-eNOS (Ser1177), AKT, P-AKT, and GAPDH. All these primary antibodies were overnight incubated at a final dilution of 1:1000. After the respective incubation period, membranes were rinsed with TBS and further incubated (1 h) with the corresponding secondary antibody at room temperature using 1:5000 dilutions. ECL western blotting substrate was used for development. Bands were quantified by densitometry using Image Studio Digits 3.1 software.

### 4.7. Glucokinase Activity

Freshly removed hepatic pieces were homogenized in handheld homogenizers (20 times) containing ice-cold phosphate saline buffer, with 0.1 mM PMSF, 0.1 mM benzamidin, 2 mM DTT, 4 μg/mL aprotinin, and 0.3 M sucrose (pH 7.5). Then, homogenates were centrifuged (600× *g*) to separate and discard the nuclear fraction. Supernatants were centrifuged (100,000× *g*, at 4 °C), collected, and identified as cytosolic fractions (where glucokinase was active). Phosphorylation in cytosolic fraction was measured at 37 °C, pH 7.4, by recording at 340 nm and increasing absorbance in a well-established enzyme-coupled photometric assay containing glucose-6-phosphate dehydrogenase, ATP, and NADP [26,45]. Glucokinase activity was obtained by subtracting the activity measured at 1 mM glucose (hexokinase) from that measured at 100 mM glucose. Enzyme activity was expressed as mU/mg of protein. One unit of enzyme activity was defined as 1 μmol of glucose-6-phosphate formed from glucose and ATP/min at 37 °C.

### 4.8. Fructokinase Activity

Pieces of liver were homogenized in a buffer containing 25 mM HEPES (pH 7.1), 100 mM KCl, 1 mM DTT, 0.1 mM EDTA, spun at 10,000× *g* at 4 °C for 20 min, and fructokinase activity was measured by a coupled enzymatic assay [46]. Briefly, 20 μL of clear supernatant was added to 200 μL of the reaction mixture [25 mM HEPES (pH 7.1), 6 mM MgCl_2_, 25 mM KCl, 10 mM NaF, 5 mM D-fructose, 0.2 mM NADH, 1 mM phosphoenolpyruvate, 40 U/mL pyruvate kinase, 40 U/mL lactate dehydrogenase, and 50 mM N-acetyl-D-glucosamine to inhibit hexokinase activity]. This reaction was started by adding 10 μL of ATP (5 mM final concentration) and quantitatively measured by recording changes in optical density at 340 nm (30 min).

### 4.9. Liver Glycogen Content

Pieces of fresh liver (400 mg) were placed in 1 mL of 33% KOH and incubated for 20 min at 100 °C. Then, 1.25 mL of ethanol was added to each tube and the mixture was incubated for 48 h at 4 °C and finally centrifuged at 700× *g* for 20 min. The pellets obtained were resuspended in 1 mL of distilled water plus 3 mL of Antrone solution (0.1% in 84% H_2_SO_4_) and incubated for 20 min at 100 °C. The absorbance was measured photometrically at 620 nm and the results expressed as μmol of glycogen/mg of tissue [47].

### 4.10. Statistical Analysis

Data were analyzed by ANOVA, followed by Tukey’s multiple comparisons test using the Prism analysis program (GraphPad 6.01). Brown–Forsythe test (sensitive to departures from normality) and Bartlett’s test were used to assess normality distribution and variance homogeneity. Results were expressed as means (±SEM) of the indicated number of observations; differences were considered significant when *p* values were <0.05.

## 5. Conclusions

The current results suggest that NAC effectively disrupts the vicious circle endocrine-metabolic alterations, oxidative stress, and inflammation triggered by sucrose. The positive effect of NAC could be ascribed to changes in the ratio of GPx/GR and, in consequence, the increase in GSH generation and on insulin signaling, AKT-dependent pathways that in turn modulated iNOS and eNOS expressions.

## Figures and Tables

**Table 1:** Serum parameters.

Parameter	Control	Sucrose	Sucrose/NAC
Glucose (mg/dL)	111.3 ± 2.87	115.9 ± 3.18	113.7 ± 1.59
Triglyceride (mg/dL)	101.1 ± 2.30	148.4 ± 8.58 *	114.3 ± 5.90 #
Insulin (ng/mL)	0.20 ± 0.004	0.27 ± 0.023 *	0.17 ± 0.016 #
HOMA-IR	1.34 ± 0.05	1.85 ± 0.18 *	1.17 ± 0.13 #
HOMA-β	13.21 ± 0.64	18.17 ± 0.28 *	10.63 ± 2.13 #
LISI	0.75 ± 0.02	0.56 ± 0.05 *	0.91 ± 0.13 #
Uric acid(mg/dL)	1.46 ± 0.15	1.40 ± 0.08	1.04 ± 0.09 #
GOT (AU/L)	18.14 ± 2.89	20.65 ± 3.87	17.68 ± 1.90
GPT (AU/L)	10.88 ± 0.63	9.16 ± 0.09	4.94 ± 0.41 #

Values are means ± SEM (n = 12). * *p* < 0.05 vs. control and # *p* < 0.05 vs. sucrose.

**Table 2:** Rat specific primers used for real-time PCR analyses. FW: forward primer, and RV: reverse primer.

Gene	GeneBank^®^	Sequences
*SREBP-1c*	XM_213329.6	FW 5′-TTTCTTCGTGGATGGGGACT-3′ RV 5′-CTGTAGATATCCAAGAGCATC-3′
*GPAT-1*	NM_017274.1	FW 5′-GACGAAGCCTTCCGAAGGA-3′ RV 5′-GACGAAGCCTTCCGAAGGA-3′
*COX-2*	NM_017232	FW 5′-GCTGCTGCCGGACACCTTCA-3′ RV 5′-CCAGCAACCCGGCCAGCAAT-3′
*iNOS*	NM_012611	FW 5′-GAAGTCCAGCCGCACCACCC-3′ RV 5′-CAGGGCCGTCTGGTTGCCTG-3′
*β-actin*	NM_031144.2	FW 5′-AGAGGGAAATCGTGCGTGAC-3′ RV 5′-CGATAGTGATGACCTGACCGT-3′

## Data Availability

Data are available on request.
